# Lipids in Regulated Exocytosis: What are They Doing?

**DOI:** 10.3389/fendo.2013.00125

**Published:** 2013-09-17

**Authors:** Mohamed Raafet Ammar, Nawal Kassas, Sylvette Chasserot-Golaz, Marie-France Bader, Nicolas Vitale

**Affiliations:** ^1^Institut des Neurosciences Cellulaires et Intégratives (INCI), UPR-3212 Centre National de la Recherche Scientifique, Université de Strasbourg, Strasbourg, France

**Keywords:** cholesterol, phosphatidic acids, phosphoinositides, exocytosis, membrane fusion, chromaffin cell

## Abstract

The regulated secretory pathway in neuroendocrine cells ends with the release of hormones and neurotransmitters following a rise in cytosolic calcium. This process known as regulated exocytosis involves the assembly of soluble *N*-ethylmaleimide-sensitive factor attachment protein receptor (SNARE) proteins, the synaptic vesicle VAMP (synaptobrevin), and the plasma membrane proteins syntaxin and SNAP-25. Although there is much evidence suggesting that SNARE proteins play a key role in the fusion machinery, other cellular elements regulating the kinetics, the extent of fusion, and the preparation of vesicle for release have received less attention. Among those factors, lipids have also been proposed to play important functions both at the level of secretory vesicle recruitment and late membrane fusion steps. Here, we will review the latest evidence supporting the concept of the fusogenic activity of lipids, and also discuss how this may be achieved. These possibilities include the recruitment and sequestration of the components of the exocytotic machinery, regulation of protein function, and direct effects on membrane topology.

## Introduction: A Role for Lipid in Exocytosis?

Release of hormones from neuroendocrine cells occurs through exocytosis, a process in which specialized vesicles (secretory granules) fuse with the plasma membrane in response to extrinsic stimuli leading to elevated cytosolic calcium. Chromaffin cells have proven to be a valuable model for unraveling the molecular mechanisms underlying this fundamental membrane fusion event (1). In these cells, secretory granules are first transported from the Golgi area to the cell periphery where they are then docked at the plasma membrane in two apparent stages: a non-primed (fusion incompetent) and a primed (fusion competent) state. Details of the molecular machinery underlying some of these steps have been described (2). For instance, once tethered to the plasma membrane, docking of granules is mediated by soluble *N*-ethylmaleimide-sensitive factor (NSF) attachment protein receptors (SNAREs) found on granules (v-SNAREs) and on the plasma membrane (t-SNAREs). Through coiled-coil interactions, these proteins form a stable complex that provides the energy necessary to pull membranes into close proximity rendering them fusion competent (3). The mechanism of membrane fusion *per se* is however an aspect that continues to be debated. SNAREs are able to drive membrane fusion *in vitro* (4), but with relatively slow kinetics suggesting that additional factors are required for physiological membrane fusion. Recently, the SNARE accessory protein Munc18 was shown to increase the speed of SNARE-mediated fusion (5). Lipids, the prime constituents of the fusing membranes, are also obvious candidates to accelerate the fusion process. The first lipid firmly demonstrated to be critical for exocytosis was phosphatidylinositol-4,5-bisphosphate [PtdIns(4,5)P_2_], for review, see Ref. (6), and more recently fatty acids have been suggested to play an important function in neurotransmitter release from synaptic vesicles (7). Growing evidence also support a role for cholesterol and phosphatidic acid (PA) during exocytosis, for review, see Ref. (8). Hence, many observations are in agreement with the notion that lipids contribute at different levels to exocytosis. The issues now arising concern the precise step in the dynamic exocytotic process in which a given lipid actually functions.

## Lipids are Involved Throughout the Sequential Stages Underlying Exocytosis

### Cholesterol and phosphatidylserine for defining exocytosis sites

Cholesterol and sphingolipids cluster into discrete microdomains in cellular membranes to form lipid ordered domains. This clustering is triggered by secretagogues at the level of exocytotic sites in chromaffin cells (9). Although cholesterol depletion by methyl-β-cyclodextrin provided the initial evidence for a positive role of cholesterol in exocytosis (10), these experiments are subjected to caution because of the brought structural role of cholesterol in most cellular functions. More compelling evidence supporting a direct role for cholesterol relies on both biochemical and high-resolution imaging observations indicating that SNAREs concentrate in cholesterol-dependent clusters (11). Additionally, a cholesterol-sequestering agent, the polyene antibiotic filipin, that is supposed to have less dramatic effects on membrane structures than methyl-β-cyclodextrin, induces a dose-dependent inhibition of catecholamine secretion and the release from the plasma membrane of annexin A2 which participates in the formation and/or stabilization of GM1-PtdIns(4,5)P_2_ enriched domains required for granule recruitment (9, 12). Altogether these findings support the notion that cholesterol is involved in the spatial definition of exocytotic sites.

In the plasma membrane, phosphatidylserine (PS) mostly resides in the inner cytoplasmic leaflet. In non-apoptotic cells, several biological functions are accompanied by a disruption of this phospholipid asymmetry resulting in the externalization of PS on the outer leaflet of the plasma membrane. This is the case for calcium-regulated exocytosis in neuroendocrine chromaffin and PC12 cells (13, 14). However the functional importance of PS scrambling for secretion is still under debate and the precise kinetics of this translocation is not established. An interesting possibility lies in the fact that PS contributes substantially to the negative charge of the inner leaflet of the plasma membrane. Consequently, PS scrambling at exocytotic sites could modify the protein/lipid interactions occurring during either the course of exocytosis or the early phases of endocytosis, as suggested recently (15).

### Phosphoinositides for priming secretory vesicles

Phosphoinositides are a class of phospholipids characterized by an inositol head group that can be phosphorylated on the three, four, and five positions to generate seven distinct species key in cell signaling and trafficking. Much of the work carried out on exocytosis has focused on the role played by PtdIns(4,5)P_2_. Indeed a number of pioneer studies indicated that PtdIns(4,5)P_2_ positively modulates secretion in neuroendocrine cells (16–18). Using patch clamp experiments on intact chromaffin cells and in parallel analyzing images of plasma membrane lawns, it was subsequently shown that over-expression of the kinase that generates PtdIns(4,5)P_2_ causes an increase in the plasmalemmal PtdIns(4,5)P_2_ level and secretion, whereas over-expression of a membrane-tagged PtdIns(4,5)P_2_ phosphatase eliminates plasmalemmal PtdIns(4,5)P_2_ and inhibits secretion (19). Thus, the balance between the generation and degradation rates of the plasmalemmal PtdIns(4,5)P_2_ directly regulates the extent of exocytosis from chromaffin cell. Using the phosphatidylinositol 3-kinase inhibitor LY294002, a correlation between the level of the plasma membrane PtdIns(4,5)P_2_ and the size of the primed vesicle pool was found (19, 20). Wen et al. (21) further demonstrated that selective inhibition of phosphatidylinositol 3-kinase delta isoform was responsible for this effect. Importantly, such an inhibition promotes a transient rise in PtdIns(4,5)P2 that was sufficient to mobilize secretory vesicles to the plasma membrane via activation of the small GTPase Cdc42 and actin polymerization. More recently, a functional link between PtdIns(4,5)P_2_ signaling and secretory vesicle dynamics through *de novo* remodeling of the actin cytoskeleton was also described (22). These observations are consistent with a function of PtdIns(4,5)P_2_ as an acute regulator of secretion. PtdIns(4,5)P_2_ seems to lie in a key position controlling the size and refilling rate of the primed vesicle pools, but not the fusion rate constants *per se*. In line with this model, we recently reported that the HIV PtdIns(4,5)P_2_-binding protein Tat is able to penetrate neuroendocrine cells and accumulate at the plasma membrane through its binding to PtdIns(4,5)P_2_. By sequestering plasma membrane PtdIns(4,5)P_2_, Tat alters neurosecretion, reducing the number of exocytotic events without significantly affecting kinetic parameters (fusion pore opening, dilatation, and closure) of individual events (23).

Other phosphoinositides seem to act as signaling or recruitment factors to prime secretory vesicles for exocytosis. For instance, experiments carried out on permeabilized chromaffin cells reveal that PtdIns(3)P located on a subpopulation of chromaffin granules positively regulates secretion (21, 24). Extending these observations, PIKfyve kinase that produces PtdIns(3,5)P_2_ from PtdIns(3)P on secretory granules was shown to negatively affect exocytosis (25). Hence, these studies highlight a complex regulation of neuroexocytosis by phosphoinositides, with PtdIns(4,5)P_2_ and PtdIns(3)P being essential factors promoting ATP-dependent priming in neurosecretory cells. It is intriguing that PtdIns(3,5)P_2_ displays an opposite effect, but reveals how fine-tuning of exocytosis by phosphoinositides could potentially control the number of vesicles undergoing priming in response to a stimulation.

### Phosphatidic acid for fusion

The local formation of PA is a recurring theme in intracellular membrane traffic and its involvement in regulated exocytosis has been suggested in various models, including neuroendocrine cells (14, 26). The development of molecular tools has enabled the identification of phospholipase D1 (PLD1) as the key enzyme responsible for PA synthesis during exocytosis (14, 27). Capacitance recordings from chromaffin cells silenced for PLD1 suggest that PLD1 controls the number of fusion competent secretory granules at the plasma membrane without affecting earlier recruitment or docking steps, leading to the idea that PA acts directly in membrane fusion (28). In agreement with this concept, a molecular sensor for PA revealed local PA accumulation at the plasma membrane near morphologically docked granules at sites of active exocytosis (28).

### Other lipids

Various other lipids are suspected to take part in regulated exocytosis. Although most of them have been implicated based on *in vitro* membrane fusion assays, some have also been studied in neuroendocrine cells. For instance, diacylglycerol (DAG) increases stimulus-coupled secretion by recruiting vesicles to the immediately releasable pool through the regulation of the vesicle priming protein Munc13-1 (29). Furthermore by activating protein kinase C, DAG may modulate the phosphorylation level of various proteins contributing or regulating the exocytotic machinery, including SNAP-25 and Munc18 (30, 31). Modulating PS levels also directly affects the rate of exocytosis in PC12 cells. Although it is likely that long term provision of high level of PS (incubation with 100 μM for 48 h or over-expression of PS synthase) may affect numerous key cellular functions altogether, it was shown that PS influences exocytosis by enhancing fusion pore opening and slowing fusion pore dilatation in PC12 cells (32). The kinetic changes in exocytosis resulting from elevated PS levels were shown to be unlikely the reflection of an indirect action of PS on Ca^2+^ channels, or vesicle size or number, or the levels of some of the major exocytosis proteins, but may be the consequence of synaptotagmin binding to PS (32). Finally, arachidonic acid produced from different phospholipids by phospholipase A2 and from DAG by DAG-lipase potentiates exocytosis from chromaffin cells (33, 34).

## Lipids as Recruiting Components of the Exocytotic Machinery

Within membranes, the ability of microdomains to sequester specific proteins and exclude others makes them ideally suited to spatially organize cellular pathways. Cholesterol-enriched microdomains seem to concentrate components of the exocytotic/fusion machinery. For instance, numerous studies of the distribution of SNARE proteins in various cell types suggest that SNAREs partially associate with detergent resistant, cholesterol-enriched microdomains (11). Palmitoylation appears to be the major targeting signal in these microdomains, as in the case of SNAP-25, although it is likely that other elements contribute to the enrichment of constituents of the exocytotic machinery within these cholesterol microdomains. However despite intense research there is still little known about what lipid or protein molecules are actually present at sites of exocytosis.

**Figure 1:**
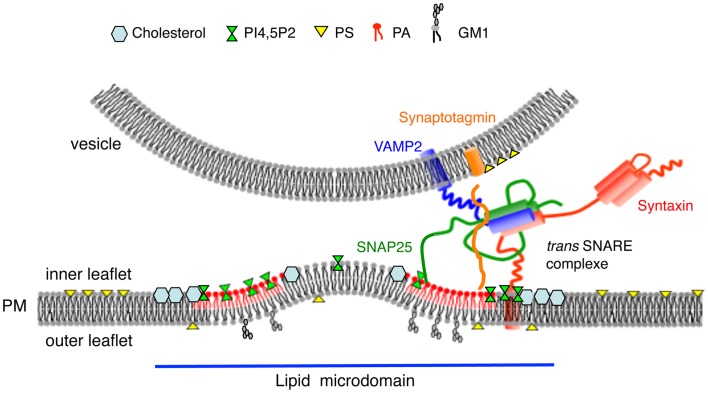
**Model highlighting the importance of lipids for membrane fusion**. Exocytotic sites defined as cholesterol, GM1, PtdIns(4,5)P_2_, and PA enriched microdomains recruit constituents of the docking/fusion machinery and create membrane curvature of the inner leaflet of the plasma membrane prior to promote membrane merging. PtdIns(4,5)P_2_ and PA may also regulate SNARE complex assembly and structure.

Up to 20 proteins potentially involved in regulated exocytosis have been reported to bind PtdIns(4,5)P_2_ (35). These include synaptotagmin (36), syntaxin (37), Ca^2+^-dependent activator protein for secretion (CAPS) (38), and members of the PLD signaling pathway (16, 39, 40). Using immunogold labeling of plasma membrane sheets combined with spatial point pattern analysis, we recently observed that PtdIns(4,5)P_2_ microdomains co-localize with SNARE clusters and docked secretory granules (12). Translocation of the PtdIns(4,5)P_2_-binding protein annexin A2 to the plasma membrane following cell stimulation is a hallmark of chromaffin cell exocytosis (41). Annexin A2 plays an essential role in calcium-regulated exocytosis by promoting PtdIns(4,5)P_2_ and cholesterol-enriched domains containing SNAREs in the vicinity of docked granules (9, 12). Altogether these observation raise the notion that functional exocytotic sites defined by specific lipids such as cholesterol, GM1, and PtdIns(4,5)P_2_ are able to recruit and sequester components to build a machine that drives fast and efficient membrane fusion (Figure 1).

Molecular details of how PtdIns(4,5)P_2_ forms a platform for vesicle recruitment have recently been proposed (42). In this study, synaptotagmin-1 was shown to interact independently of Ca^2+^ with the polybasic linker region of syntaxin-1A already associated with PtdIns(4,5)P_2_ at the plasma membrane. When then triggered by Ca^2+^, this interaction allows the Ca^2+^-binding sites of synaptotagmin-1 to bind PS in the vesicle membrane, thereby bridging the vesicle membrane close enough for SNARE assembly and subsequent membrane fusion. Interestingly, this polybasic juxtamembrane domain of syntaxin-1A which binds PtdIns(4,5)P_2_ has also been shown to tightly bind both PA (37) and PtdIns(3,4,5)P_3_ (43). Like PtdIns(4,5)P_2_, these anionic lipids can probably recruit syntaxin-1A, and it is tempting to propose that the recruitment of syntaxin isoforms may depend on the type of lipid present. Furthermore, the fact that these lipids can be quickly converted into different forms using kinases, lipases, and phosphatases, such a protein-recruiting mechanism offers a supplementary level of control to adapt the exocytotic machinery to the physiological demands put on the cell. Finally, using super-resolution optical techniques and fluorescence lifetime imaging microscopy, it was shown that distinct t-SNARE intermediate states on the plasma membrane can be patterned by the underlying lipid environment (44). Undoubtedly these high-resolution imaging techniques will be useful to determine how lipids contribute to the organization of the exocytotic platform.

## Lipids as Regulators of the Exocytotic Machinery

PtdIns(4,5)P_2_ directly binds to a large subset of proteins from the exocytotic machinery. *In vitro* experiments revealed that at physiological concentrations PtdIns(4,5)P_2_ regulates exocytosis by recruiting priming factors such as CAPS, which facilitate SNARE-dependent liposome fusion (45). PtdIns(4,5)P_2_ is also a necessary cofactor for PLD1 activity and PA synthesis during exocytosis (39). Finally, PtdIns(4,5)P_2_ controls actin polymerization by modulating the activity and targeting of actin regulatory proteins. Indeed the activity of the actin-binding proteins scinderin and gelsolin, two F-actin severing proteins that are constituents of the exocytotic machinery, is regulated by PtdIns(4,5)P_2_ (46). A transient increase in PIP2 levels is sufficient to promote the mobilization and recruitment of secretory vesicles to the plasma membrane (22). PIP2 therefore links exocytosis and the actin cytoskeleton by coordinating the actin-based delivery of secretory vesicles to the exocytotic sites.

Diacylglycerol production through hydrolysis of PtdIns(4,5)P_2_ by phospholipase C is mandatory for exocytosis (47). DAG is essential in the priming of exocytosis, owing to the activation of protein kinase C and Munc13, which then modulate the function of syntaxin-1A (29). It is worth to mention that up to 10 different DAG-kinases may also produce DAG from various subcellular pools of PA during exocytosis. Finally DAG is further hydrolyzed by DAG lipases to liberate fatty acids and monoacylglycerols. This pathway is essential for exocytosis as inhibition of DAG lipase blocks exocytosis (48).

Several constituents and regulators of the exocytotic machinery have also been shown to bind to PA, including small GTPases, NSF, and syntaxin-1A (49). PA directly activates these proteins, but evidence that this activation directly contributes to exocytosis remains scarce. PA is also an essential cofactor of phosphatidylinositol-4-phosphate 5-kinase, which produces PtdIns(4,5)P_2_, suggesting a possible positive feedback loop in the synthesis of PA and PtdIns(4,5)P_2_ (50). Although no direct evidence in neuroendocrine systems have shown that PA directly regulates the assembly or the function of the minimal fusion machinery, *in vitro* reconstituted fusion assays with purified yeast vacuolar SNAREs do so. Indeed experiments performed in a complex reconstituted system including the SNARE chaperones Sec17p/Sec18p, the multifunctional HOPS complex including a subunit of the Sec1-Munc18 family and vacuolar lipids suggest that PA is equally essential for SNARE complex assembly and for fusion. PA has been proposed to facilitate functional interactions among SNAREs and SNARE chaperones (Figure 1). Interestingly, in this system, PA could not be replaced by either lipids with small headgroups, such as DAG or acidic lipids, like PS or PI (51).

Arachidonic acid has been described to directly promote the assembly of syntaxin-3 with SNAP-25 and the formation of the ternary SNARE complex (52). Interestingly, omega-3 and omega-6 fatty acids, which play important roles in human health, have be shown to recapitulate this *in vitro* effect of arachidonic acid on SNARE complex formation, suggesting that syntaxins may represent crucial targets of polyunsaturated lipids (52). In other words, polyunsaturated lipids may physiologically regulate SNARE complex assembly and thus exocytosis. Along this same line, sphingosine a releasable backbone of sphingolipids, activates vesicular synaptobrevin facilitating the assembly of SNARE complexes required for membrane fusion (53). It is however important to note that the effects of arachidonic acid and sphingosine observed in these studies are all achieved near or at the CMC value for these lipids, treatments that may also lead to membrane disorganization like detergent action.

## Lipids Affect Membrane Topology during Membrane Fusion: The Concept of Fusogenic Lipids

The most widely accepted model for membrane fusion, the stalk pore model proposes that the merging of *cis* contacting monolayers gives rise to a negatively curved lipid structure called a stalk. The structure of this stalk depends on the composition of the *cis* monolayers (the outer leaflet of the vesicle and the inner leaflet of the plasma membrane). This model implies that cone-shaped lipids such as cholesterol, DAG, or PA, which have intrinsic negative curvatures, in the *cis* leaflets of contacting bilayers would enhance membrane fusion (54). *Vice versa* inverted cone-shaped lipids (such as PS, gangliosides, or lysophospholipids) should prevent membrane fusion in the *cis* leaflets, but promote fusion when present in the outer leaflets (54). Interestingly, GM1 was found enriched in the outer leaflet of the plasma membrane at the sites of exocytosis in stimulated chromaffin cells (9). These GM1 domains may induce positive membrane curvature in the outer leaflet (55), thereby promoting fusion (Figure 1). Reconstituted fusion assays and direct addition of lipids on cultured cells validate the concept that PA, DAG, and cholesterol might promote membrane fusion by changing the spontaneous curvature of membranes [reviewed in (8)]. At physiological concentrations, PtdIns(4,5)P_2_ inhibits SNARE-dependent liposome fusion (45), most likely due to its intrinsic positive curvature-promoting properties. However, PtdIns(4,5)P_2_ has been described to be converted from an inverted cone-shaped structure to a cone-shaped form in the presence of calcium (56). Thus, in stimulated cells, a local accumulation of PA and PtdIns(4,5)P_2_ at granule docking sites where GM1 is in the outer leaflets may well have a synergistic effect on membrane curvature and promote fusion (Figure 1). In an alternate mode of changing membrane topology, synaptotagmin has been proposed to facilitate membrane fusion by phase separating PS, a process that is expected to locally buckle bilayers and disorder lipids due to the curvature tendencies of PS (57). It is worth to mention that most of lipid mentioned in this review, also have the ability to flip from one leaflet to another. How this flipping is regulated and how it affects curvature remains an unsolved issue. However, it is likely that the ability of these lipids to interact with the fusion machinery largely controls these flipping properties.

## Conclusion

As genuine components of the exocytotic machinery, lipids have several advantages over proteins for this task: lipids can directly change the intrinsic fusion properties of membranes, recruit, and/or activate a large number of different proteins to create a local environment in which exocytosis takes place (Figure 1). As illustrated in this review, a given lipid can play multiple functions, acting either individually or successively or even simultaneously in concert with other lipids. At the same time, the rapid enzymatic production and degradation of lipids at exocytotic sites allows the cell to remain flexible: by changing the lipid levels, physiological function can be modified within seconds or minutes without the need for protein synthesis or degradation. Over the last decade, *in vitro* reconstituted membrane fusion combined with precise methods to quantify specific lipid species and improved molecular and pharmacological tools to manipulate cellular levels of a given lipid, have lead to a better understanding of the capacities of lipids to promote exocytosis at different steps of the process. For different kinds of vesicles in different cell types, it is likely that the local lipid environment may differentially regulate fusion pore formation, enlargement, and duration, which may in part explain the great variety of fusion kinetics observed *in vivo*. Finally, lipids could also contribute to the tight coupling between exocytosis and the early stages of membrane retrieval and endocytosis as highlighted in a review of this issue (Houy et al., submitted). Undoubtedly, the next challenge will be to follow individual lipid dynamics at the speed of pore formation and expansion.

## Conflict of Interest Statement

The authors declare that the research was conducted in the absence of any commercial or financial relationships that could be construed as a potential conflict of interest.
